# Nutritional status and high adherence to the Mediterranean diet in Colombian school children and teenagers during the COVID-19 pandemic according to sex

**DOI:** 10.1017/jns.2021.48

**Published:** 2021-07-13

**Authors:** William Javier Morales Camacho, Sonia Esperanza Osma Zambrano, María Alejandra Morales Camacho, Angie Carolina Herrera Contreras, Angela Rangel Acevedo, Edgar Julián Duarte Valencia, Anamaria Camargo Cárdenas, Laura Ximena Nocua Alarcón, Lizeth Carolina Ardila Munar, Ana Milena Noguera Sánchez, Jorge Mario Molina Díaz

**Affiliations:** 1 Investigation Group of Pediatrics El Bosque University (UEB), Bogotá D.C., Colombia; 2 Universidad Autónoma de Bucaramanga (UNAB), Bucaramanga, Santander; 3 Antonio Nariño University (UAN), Bogotá D.C., Colombia; 4 University of Cartagena (UDC), Cartagena, Bolívar, Colombia; 5 Pontifical Javeriana University, Bogotá D.C., Colombia; 6 Department of Child Endocrinology, Federico Gómez Children's Hospital of Mexico (HIMFG), Ciudad de México, Mexico; 7 Autonomous University of Mexico (UNAM), Mexico City, Mexico

**Keywords:** Diet, Child nutrition disorders, Coronavirus infections, Mediterranean, Paediatric obesity

## Abstract

The current COVID-19 pandemic has generated a series of changes in the daily routines of people, including children and teenagers, in an unprecedented way, which constitutes a global challenge in public health. Social isolation has been a prophylactic measure to prevent the spread of the virus; however, it has generated negative impacts on the physical and emotional health of parents, caregivers, children and teenagers around the world. The objective of the present study was to evaluate the effects of confinement caused by the COVID-19 pandemic at the level of nutritional status, dietary and behavioural patterns of elementary school children and teenagers in a small town of Colombia. Anthropometric parameters such as BMI *Z*-score, waist circumference and waist/height ratio were evaluated in 266 school children and teenagers. A questionnaire with socio-demographic, clinical and lifestyle characteristics and the KIDMED were applied to learn about nutritional aspects. A total of 102 students (38⋅3 %) were classified as having altered nutritional status, being 39 (14⋅7 %) classified as overweight and 36 (13⋅5 %) with obesity. The prevalence of high adherence to the Mediterranean diet was 12 %, 95 % CI (0⋅08, 0⋅16). Overweight was more prevalent in women (26/39, 66⋅7 %; *P* = 0⋅0439), and obesity was discreetly more frequent in men (19/36, 52⋅7 %; *P* = 0⋅7193). We observed a worrying nutritional, dietary and behavioural situation in the children and teenagers studied during the confinement associated with the COVID-19 pandemic. This unveils the need to establish strategies and/or public policies in our town that help to promote an adequate biopsychosocial development of the paediatric patient and their family group.

## Introduction

The current COVID-19 pandemic has generated a series of changes in the daily routines of people, including children and teenagers, in an unprecedented way^(1,2)^. From this perspective, many factors influence negatively the physical and mental health of children and teenagers. They experience the stress inherent in the pandemic, such as the isolation itself, school closings, reduced social life and physical activities in parks, gyms or leisure areas, changes in routine, sleeping difficulties, exposure to disharmony at home, excessive use of screens and an unhealthy diet due to the excessive consumption of industrialised foods^(3–5)^. Nutritional risks in children must be evaluated because nutritional alterations (child malnutrition, overweight or obesity) are accentuated in the most vulnerable population sectors^(3–5)^. This is due to aspects such as a decrease in public support in these sectors (closures or restrictions of schools, nongovernmental organisations or restaurants that provide food) with consequent difficulties in healthy nutrition^(4,5)^. Therefore, it is important to evaluate and identify the factors related to the pandemic that negatively affect the growth and development of children and teenagers, so that prevention strategies can be developed^(4,5)^. These strategies should have an impact on the reduction of potential losses in individual and collective health, and the long-term deterioration of the cognition, physical and mental health, and work capacity of future adults^(4,5)^. The present study aims to evaluate the effects of confinement caused by the COVID-19 pandemic at the level of nutritional status, dietary and behavioural patterns of children and teenagers from elementary school in a small town of Colombia. The Mediterranean diet was taken as a nutritional reference. It is considered a model of a balanced and healthy diet due to its composition rich in vegetables, fruits, legumes, cereals and antioxidants^(6,7)^ that modulate pro-inflammatory cytokines such as interleukin (IL) 6, IL-1, IL-2 and Tumor Necrosis Factor (TNF) α^7^, and it has been associated with a lower frequency of preventable chronic diseases and some types of cancer^(6–14)^.

## Materials and methods

An observational, analytical, prolective cross-sectional study was carried out with 266 children who attended one of the seven different elementary schools in the town called Santa Rosa del Sur, Colombia. A sample size of 290 was calculated using an assumed prevalence of childhood malnutrition and overweight/obesity of 0⋅31, with a 5 % margin of error at a 95 % confidence level (CI). Prior to the randomisation process, eight students were excluded due to eligibility criteria. The study response rate was 91⋅7 %. A stratified random sampling was performed, and the total number of students included from each institution was directly proportional to the total number of students of each of them. The eligibility criteria were to attend any of the basic primary grades in one of the seven educational institutions that are present there, not having a known disease, the absence of routine use of drugs to control underlying pathologies, and having a parent available to complete the proposed questionnaire. The data were collected after 4 months of mandatory confinement associated with the COVID-19 pandemic during the months of July and August 2020, in accordance with the Declaration of Helsinki and its subsequent modifications. The María Montessori school provided the ethical approval for this research work, informed assent and consent were obtained from the children and parents, respectively, for the taking of anthropometric measurements, completion of the questionnaire and anonymous registration of the data. Only the principal and the co-investigators who collected the data knew the identity of the children.

### Anthropometric measurements and evaluation of fat mass

A team made up of six general practitioners and two undergraduate medical students filled out the questionnaires to learn about the socio-demographic, nutritional and lifestyle aspects of each child. A paediatric specialist collected the anthropometric variables such as weight, height and waist circumference (CC) using standardised methods. Weight (kg) and height (cm) measurements were taken with the child in light clothing, without shoes on and without accessories such as hair ornaments, braids or hats and socks. A portable scale (Health O Meter 844kl), a stadiometer (Seca 222 ®) and a flexible non-elastic measuring tape were used. Initially, two measurements were made: for the weight reading, the closest 0⋅1 kg was considered, and for the height, the closest 0⋅1 cm was taken into account. Also, the closest 0⋅1 cm for the WC was considered. WC was recorded after the children gently exhaled, and the measurement was made at the narrowest part between their lower rib and the iliac crest (natural waist). If there was a difference between the two corresponding anthropometric measurements, a third measurement would be taken and averaged.

BMI, BMI *Z*-score and waist-to-height ratio (WtHR) were calculated as previously reported. Children were classified as thin or at risk of thinness, normal weight, overweight and obesity according to the age- and sex-specific International Obesity Task Force (IOTF)^(15)^.

### Questionnaires

The instruments used included a socio-demographic characterisation questionnaire, the KIDMED^(16)^ and two questions related to changes in behaviour during confinement. The first information collection format (ICF) included variables such as sex, age of the child, age and profession/occupation of the parents, composition of the household and nutritional status of relatives within the household. The questionnaire also inquired about the characteristics and behaviours of the child related to sleep pattern, the practice of physical activity and the hours dedicated to leisure (television, telephone and computer measured in minutes per day). Additionally, an open self-assessment question was asked to estimate the parents’ perception of their child's real weight: ‘In your opinion, is your son/daughter in: underweight, normal weight, overweight or obesity?’

KIDMED^(16)^ is a validated scale, made up of sixteen questions, which aims to analyse the consumption and the frequency of daily intake of various foods. This instrument was originally developed to assess the level of adherence to MD in Spanish children and young people from 2 to 24 years of age. The sum of their values varies between 0 and 12 points, and it allows classifying adherence to MD as follows: high (≥8 points), moderate (4–7 points) and low adherence (≤3 points). The version used in the present study was adapted from the version by Serra-Majem *et al.*^(16)^.

### Statistic analysis

In the univariate statistical analysis, the qualitative variables with absolute frequencies and percentage, and the quantitative variables with mean and standard deviation were described. The nutritional status was established in four categories according to the *Z*-score of the BMI. To complement, the perception of weight by the parents was described, and a variable was formed with the level of agreement between the real weight and the weight estimated by the parents, and it was established whether the nutritional status of their children is overestimated or underestimated (Table 3).

Differences were established between all variables, including nutritional status with high adherence to the MD considering the dichotomous variable, collapsing low and moderate with high adherence according to sex using Pearson's Chi-square test (*χ*^2^) or Fischer's exact test or student's *t*. 95 % confidence intervals were calculated. To explain the high adherence to the MD, we included sex, categorised age (<9 years: ≥9 years), high abnormal weight (thin and normal weight: overweight and obesity), having received breastfeeding for 12 months (<12 months: ≥12 months), categorised mother and father education, father and brother overweight and lifestyle variables using logistic regression. Differences were established considering as significant a value of *P* < 0⋅05. The processing and analysis of the information was carried out in the statistical program Stata 14.

## Results

Table 1 shows the socioeconomic, anthropometric, nutritional and lifestyle characteristics of the children grouped according to sex. In total, 266 elementary school children between 5⋅1 and 14⋅8 years old were evaluated, including 142 (53⋅4 %) women and 124 (46⋅6 %) men. The mean age of the participants was 8⋅5 ± 2⋅04 years. In relation to nutritional status, a total of 164 (61⋅7 %) students were classified as having adequate nutritional status (eutrophic), while 102 were considered to have altered nutritional status, including 27 (10⋅1 %) in thinness or risk of thinness, 39 (14⋅7 %) with overweight and 36 (13⋅5 %) with obesity. The 50th percentile of BMI of the participants was 56⋅8 ± 31⋅3. Thinness or risk of thinness was more prevalent in children (*n* 17/27, 62⋅9 %; *P* = 0⋅1746). Women were twice as overweight (26/39,⋅66⋅7 %) compared to men (13/39,⋅33⋅3 %; *P* = 0⋅0439). Likewise, obesity was slightly more frequent in men (*n* 19/36, 52⋅7 %; *P* = 0⋅7193).

**Table 1.** Demographic and anthropometric characteristics, nutritional status and lifestyles according to sex

	Total	Male	Female	*P*-value
*N*	266	124 (46⋅6 %)	142 (53⋅4 %)	
Age	8⋅5 ± 2⋅04	8⋅4 ± 2⋅0	8⋅7 ± 2⋅0	0⋅4617
Height	119⋅2 ± 12⋅6	134 ± 12	111⋅9 ± 13⋅2	0⋅7505
Weight	28⋅8 ± 7⋅5	21⋅8 ± 4⋅8	32⋅6 ± 9⋅2	0⋅3220
Correctly classified	136 (51⋅1 %)	61(49⋅2 %)	75(52⋅8 %)	0⋅6426
50th percentile BMI	56⋅8 ± 31⋅3	57⋅9 ± 32⋅1	55⋅8 ± 30⋅7	
Overweight	39 (14⋅7 %)	13 (33⋅3 %)	26 (66⋅7 %)	0⋅0439
Obesity	36 (13⋅5 %)	19 (52⋅8 %)	17 (47⋅2 %)	0⋅7193
Central obesity	86 (32⋅3 %)	37 (43⋅0 %)	49 (57⋅0 %)	0⋅1985
Thinness or risk of thinness	27(10⋅1 %)	17 (62⋅9 %)	10 (36⋅1 %)	0⋅1746
Risk of delayed height or short stature for age	76 (28⋅5 %)	33 (43⋅4 %)	43 (56⋅6 %)	0⋅2612
KIDMED				
Low adherence	70 (26⋅3 %)	28 (0⋅4 %)	42 (0⋅6 %)	0⋅1008
Medium adherence	164 (61⋅6 %)	88 (53⋅6 %)	76 (46⋅3 %)	0⋅3069
High adherence	32 (12⋅1 %)	8 (25 %)	24 (75 %)	0⋅0114
Frequency of physical activity/week				
<3 times	86 (32⋅4 %)	34 (39⋅5 %)	52 (60⋅4 %)	0⋅4007
≥3 times	169 (63⋅5 %)	86 (50⋅8 %)	83 (49⋅1 %)	0⋅8887
Not done	11 (4⋅1 %)	4 (36⋅3 %)	7 (63⋅6 %)	0⋅9117
Hours of exercise per week				
<3 h	198 (74⋅4 %)	92 (46⋅5 %)	106 (53⋅5 %)	0⋅4690
≥3 h	68 (25⋅6 %)	32 (47⋅0 %)	36 (53⋅0 %)	0⋅8070
Screen hours/day				
<3 h	242 (91 %)	128 (48⋅1 %)	114 (42⋅9 %)	0⋅3514
≥3 h	24 (9 %)	14 (5⋅3 %)	10 (3⋅7 %)	0⋅4394

Abdominal obesity (waist/height index ≥ 0⋅5) was found in 32⋅3 %, 95 % CI (0⋅27, 0⋅38) of the participants, and it was more prevalent in women (49/86, 57 %) compared to men (37/86, 43⋅0 %; *P* = 0⋅417), also corresponding to 14 (87⋅5 %) in overweight condition and 11/25 (44 %) in obesity condition.

The analysis of nutritional habits showed that only 12⋅1 %, 95 % CI (0⋅09, 0⋅16) of the participants had high adherence to the MD, 61⋅6 and 26⋅3 % of them had moderate and low adherence, respectively.

During the confinement period, it was found that 65⋅8 % of the participants reported changes in behaviour, predominantly irritability and hyperactivity (40 % *v.* 27⋅4 %, respectively). There were no statistically significant differences in behaviour according to the sex of the participants (*P* = 0⋅2360).

Table 2 shows the responses obtained in the KIDMED questionnaire according to gender and the total sample. We found very similar responses according to the gender of the participants. Among the positive results, we highlight the habit of having breakfast every day as well as the consumption of legumes more than once a week, and consumption of pasta or rice almost daily (5 d or more a week). In turn, we highlight a low number of responses in the items that inquire about the consumption of nuts at least two or three times a week and the use of olive oil. The responses to each item were analysed by gender to verify associations between these variables. No statistically significant differences were found between the variables of the KIDMED questionnaire and gender.

**Table 2.** Responses obtained in the questionnaire of adherence to the Mediterranean diet according to gender

KIDMED	Total (*n* 266)	Male (*n* 124)	Female (*n* 142)	*P*-value
	%	%	%	
1. Do you eat at least one piece of fruit every day?	71⋅4	71⋅7	71⋅1	0⋅907
2. Do you eat more than one piece of fruit every day?	27	23⋅3	30⋅2	0⋅207
3. Do you regularly (4 or more days a week) eat vegetables, either raw (lettuce, tomato, etc.) or cooked (broccoli, cabbage, etc.), once a day?	56⋅3	54	58⋅4	0⋅469
4. Do you regularly (4 or more days a week) eat raw or cooked vegetables more than once a day?	29⋅3	30⋅6	28⋅1	0⋅658
5. Do you eat fish regularly (at least twice a week)?	41⋅3	43⋅5	39⋅4	0⋅497
6. Do you go to a fast-food restaurant more than once a week?	27⋅8	29	26⋅7	0⋅680
7. Do you like legumes (beans, grains, peas, etc.) and have them more than once a week?	93⋅6	94⋅3	92⋅9	0⋅642
8. Do you eat pasta or rice almost every day (5 or more days a week)?	96⋅9	95⋅9	97⋅8	0⋅657
9. Do you eat cereals and their products (bread, breakfast cereals) for breakfast?	64⋅2	64⋅5	64	0⋅942
10. Do you eat dried fruits (nuts, hazelnuts, etc.) regularly (at least two to three times a week)?	10⋅1	6⋅4	13⋅3	0⋅543
11. Do you use olive oil in your home?	12⋅8	13⋅7	11⋅9	0⋅672
12. Do you usually (4 or more days a week) have breakfast?	92⋅8	93⋅5	92⋅2	0⋅682
13. Do you have a dairy product (yoghurt, milk, etc.) for breakfast?	68	69⋅3	66⋅9	0⋅751
14. Do you eat pastry/confectionery products for breakfast?	27	22⋅5	30⋅9	0⋅124
15. Do you eat two yoghurts and/or a slice of cheese (40 g) daily?	27⋅8	33	23⋅2	0⋅074
16. Do you eat candies and snacks several times a day?	40⋅2	39⋅5	40⋅8	0⋅825

KIDMED, Mediterranean Diet Quality Index in Children and Adolescents.

Table 3 shows the perception of parents about the weight of their children and its corresponding interpretation according to the real weight. In 51⋅1 % of the participants, there was an adequate correlation between the perception of weight of the parents and the actual weight of the child, an underestimation of the child's weight was observed in 38 % (*n* 101) and an overestimation in 10⋅9 % (*n* 29) of the participants.

**Table 3.** Parental perception of children's weight status

Correct [*n* 136 (51⋅1 %)]			Underestimation [*n* 101 (38 %)]			Overestimation [*n* 29 (10⋅9 %)]		
Real	Perception	*n* (%)	Real	Perception	*n* (%)	Real	Perception	*n* (%)
Underweight	Underweight	14 (5⋅3)	Obesity	Overweight	16 (6⋅0)	Underweight	Normal	12 (4⋅5)
Normal	Normal	108 (40⋅6)	Overweight	Normal	21 (7⋅9)	Normal	Overweight	15 (5⋅7)
Overweight	Overweight	11 (4⋅1)	Normal	Underweight	40 (15)	Overweight	Obesity	0 (0)
Obesity	Obesity	3 (1⋅1)	Obesity	Normal	13 (4⋅9)	Underweight	Overweight	1 (0⋅35)
			Obesity	Underweight	4 (1⋅5)	Normal	Obesity	1 (0⋅35)
			Overweight	Underweight	7 (2⋅7)			

In relation to the factors associated with having high adherence to the MD, the bivariate analysis found significant differences in sex OR 0⋅34 (0⋅15, 0⋅78; *P* = 0⋅012) and categorised age: OR 0⋅35 (0⋅15, 0⋅81; *P* = 0⋅015). Finally, in the multivariate model constructed with having been breastfed for >12 months, having a high abnormal weight, categorised age and sex, it was found that being less than 9 years old is associated with high adherence to the MD (OR 0⋅32; 0⋅14, 0⋅75; *P* = 0⋅009), and being a man increases 3⋅2 times more the chance of having low or moderate adherence to the MD compared to women (OR 0⋅31; 0⋅14, 0⋅75).

## Discussion

The present study aimed to evaluate the effects of confinement caused by the COVID-19 pandemic at the level of nutritional status, dietary and behavioural patterns of children and teenagers from elementary school in a small town of Colombia. In this sense, the prevalence rates of the double burden of disease (malnutrition, overweight/obesity) found in the present study generate great concern regarding the health of children and teenagers in Santa Rosa del Sur, Colombia during the current COVID-19 pandemic. The present study estimates a prevalence of underweight, overweight and obesity among school children and teenagers of 10⋅1, 14⋅7 and 13⋅5 %, respectively, and these numbers that turn out to be higher compared to those reported in the last national health survey in Colombian children (Ensin2015)^(17)^ with prevalences of underweight and overweight of 7 and 24⋅4 %, respectively. Compared with other studies, prevalence rates were similar to those previously reported in children and teenagers in Europe^(18–21)^, Africa^(22)^, Asia^(23,24)^ and South America^(25,26)^. However, we estimate higher proportions of both, obesity measured by BMI and central obesity, than those reported in other studies^(6,23,24,26–33)^. The prevalence of high abnormal weight was 28⋅2 % similar to that reported by Garrido *et al.* where it is described that 32⋅1 % of young people aged 7–13 years were overweight and obese^(34)^.

The overweight ratio was two women for every man and obesity was slightly more frequent in men (19/36, 52⋅7 %). These findings contrast with those previously described in Asian^(31,35)^ and European^(6,18–21)^ children, but they are similar to those reported in 2018 by Silva *et al.* in the state of Minas Gerais, Brazil^(25)^.

With regard to sedentary and nutritional habits and despite the fact that 74⋅4 % of the participants in our studies performed <3 h of physical activity per week, no statistically significant differences were found in the nutritional status of the patients. This is a finding that is consistent with the one reported by Smetanina *et al.* in Lithuania^(18)^, but it is different from what was reported in Brazil^(25,36,37)^, Mexico^(38)^, Australia^(39)^, Egypt^(40)^, Nigeria^(33)^, Nepal^(31)^, Iran^(41)^ and among others^(23,42)^. The relationship between the educational level of the parents and the nutritional status of the children continues to be contradictory, because some studies have reported higher frequencies of overweight or obesity in children whose parents have a higher educational level^(25,31,35,43)^, yet, other studies associate less education with higher rates of overweight/obesity^(18)^. In our particular case, we did not find significant differences in such variables.

Regarding the second objective, adherence to the MD was low in 26⋅3 %, moderate in 61⋅6 % and high in 12⋅1 % of the participants, in discrepancy with what was reported by Santos Marques *et al.* in two villages in Portugal (Porto and Maia), in whose study describes a high adherence to MD in 77⋅6 % of the participants^(44)^. These differences may be due to the urban environment of these cities and their concern for the promotion of healthy lifestyles, which is different from the rural environment and the lack of clear and well-defined public policies in our case. Furthermore, our results also disagree with different studies in the European population with low adherence between 16⋅7 and 18⋅5 %, and high between 19⋅6 and 29⋅3 % of the participants^(6,45,46)^. No significant differences were found between adherence to the MD and the BMI of the participants, so it should be noted that such an adherence does not guarantee adequate nutritional status by itself, since we would be leaving aside the synergistic role that healthy habits can exert, such as physical activity and sedentary time^(46)^. We found that age under 9 years and female sex had a statistically significant association with high adherence to the MD, although our results are in contrast to those described by Novak *et al.*^(47)^ in European teenagers, this could be due to the fact that both school children and teenagers were included in the study, and their hypothesis was valid that during adolescence, women were more sedentary because of their school obligations accompanied by stress and irregular eating. This would suggest that the protective factor found in the present study dissipates in as much as there are more academic and/or work responsibilities acquired by women, which would explain the higher prevalence of overweight/obesity in females, mainly after the age of 15^(48)^.

Finally, we found another important concern in the present study, and it is a wrong perception of parents about the weight of their children in 48⋅9 % of the cases (underestimation: 38 %, overestimation: 10⋅9 %). This is a higher percentage compared to what was reported by Sirico *et al.* in Italian parents^(45)^, which suggests a poor awareness in general regarding childhood overweight/obesity, and medium and long-term complications in parents both in the study by Sirico *et al.* and in the present study. This can be linked to various sociocultural factors, as well as to their educational level, and the psychology and economy of the parents^(45,49)^.

All things considered, however, reveal that the present study has some limitations. First, physical activity and screen times were not measured objectively, but through a questionnaire given to parents. Secondly, the data correspond to school children and teenagers in a town in Colombia, so the results cannot be extrapolated to the general Colombian population. Finally, since it is a descriptive and cross-sectional study, cause and effect relationships cannot be established between the study variables. Despite the limitations, the work has great strength, since it provides updated and representative information that represents a starting point from the nutritional approach of the paediatric patient during the current COVID-19 pandemic.

In conclusion, we observed a worrying nutritional, dietary and behavioural situation in the children and teenagers studied during the confinement associated with the COVID-19 pandemic. This unveils the need to establish strategies and/or public policies in our town that help to promote an adequate biopsychosocial development of the paediatric patient and their family group, having as fundamental pillars a healthy diet and adequate physical activity.

## References

[1] Singh S, Roy D, Sinha K, (2020) Impact of COVID-19 and lockdown on mental health of children and adolescents: a narrative review with recommendations. Psychiatry Res 293, 113429. 32882598 10.1016/j.psychres.2020.113429 PMC7444649

[2] Nicola M, Alsafi Z, Sohrabi C, (2020) The socio-economic implications of the coronavirus pandemic (COVID-19): a review. Int J Surg 78, 185–193. 32305533 10.1016/j.ijsu.2020.04.018 PMC7162753

[3] Eisenstein E, Estefenon S, Gama MC, (2020) Recomendações sobre o uso saudável das telas digitais em tempos de pandemia da COVID-19 # BOAS TELAS # MAIS SAÚDE. Soc Bras Pediatr (21 de maio), 1–5. https://www.sbp.com.br/fileadmin/user_upload/22521b-NA_Recom_UsoSaudavel_TelasDigit_COVID19__BoasTelas__MaisSaude.pdf

[4] Araújo LA, Veloso CF, Souza MC, (2020) The potential impact of the COVID-19 pandemic on child growth and development: a systematic review. J Pediatr (Rio J). S0021-7557(20)30209-6. 10.1016/j.jped.2020.08.008 PMC7510529 32980318

[5] Wang G, Zhang Y, Zhao J, (2020) Mitigate the effects of home confinement on children during the COVID-19 outbreak. Lancet 395, 945–947. 10.1016/S0140-6736(20)30547-X PMC7124694 32145186

[6] Archero F, Ricotti R, Solito A, (2018) Adherence to the Mediterranean diet among school children and adolescents living in Northern Italy and unhealthy food behaviors associated to overweight. Nutrients 10, 1322. 10.3390/nu10091322 PMC6165180 30231531

[7] Carvalho KMB, Ronca DB, Michels N, (2018) Does the Mediterranean diet protect against stress-induced inflammatory activation in European adolescents? The HELENA study. Nutrients 10, 1770. 10.3390/nu10111770 PMC6266959 30445703

[8] Esposito K & Maiorino MI (2015) A journey into a Mediterranean diet and type 2 diabetes: a systematic review with meta-analysis. BMJ Open 5, e008222. 10.1136/bmjopen-2015-008222 PMC4538272 26260349

[9] Mattioli AV & Palmiero P (2017) Mediterranean diet impact on cardiovascular diseases: a narrative review. J Cardiovasc Med 18, 925–935. 10.2459/JCM.0000000000000573 28914660

[10] Schwingshackl L & Schwedhelm C (2017) Adherence to Mediterranean diet and risk of cancer: an update systematic review and meta-analysis. Nutrients 9, 1063. 10.3390/nu9101063 PMC5691680 28954418

[11] Grosso G & Marventano S (2017) A comprehensive meta-analysis on evidence of Mediterranean diet and cardiovascular disease: are individual components equal? Crit Rev Food Sci Nutr 57, 3218–3232. 26528631 10.1080/10408398.2015.1107021

[12] Arouca AB, Santaliestra-Pasías AM, Moreno LA, (2019) Diet as a moderator in the association of sedentary behaviors with inflammatory biomarkers among adolescents in the HELENA study. Eur J Nutr 58, 2051–2065. 29974229 10.1007/s00394-018-1764-4

[13] Smidowicz A & Regula J (2015) Effect of nutritional status and dietary patterns on human serum C-reactive protein and interleukin-6 concentrations. Adv Nutr 6, 738–747. 26567198 10.3945/an.115.009415 PMC4642421

[14] Donini LM, Serra-Majem L, Bulló M, (2015) The Mediterranean diet: culture, health and science. Br J Nutr 113, S1–S3. 10.1017/S0007114515001087 26148911

[15] Cole T, Bellizzi M, Flegal K, (2000) Establishing a standard definition for child overweight and obesity worldwide: international survey. Br Med J 320, 1240–1243. 10797032 10.1136/bmj.320.7244.1240 PMC27365

[16] Serra-Majem L & Ribas L (2004) Food, youth and the Mediterranean diet in Spain. Development of KIDMED, Mediterranean diet quality index in children and adolescents. Public Health Nutr 7, 931–935. 15482620 10.1079/phn2004556

[17] ENSIN, Encuesta Nacional de la Situación Nutricional (2015). Ministerio de Salud, Departamento para la Prosperidad Social y ICBF [Internet]. Gov.co. [cited 2021 Jun 29]. Available at https://www.icbf.gov.co/bienestar/nutricion/encuesta-nacional-situacion-nutricional.

[18] Smetanina N, Albaviciute E, Babinska V, (2015) Prevalence of overweight/obesity in relation to dietary habits and lifestyle among 7-17 years old children and adolescents in Lithuania. BMC Public Health 15, 1001. 26429124 10.1186/s12889-015-2340-y PMC4590263

[19] Birbilis M, Moschonis G, Mougios V, (2012) Obesity in adolescence is associated with perinatal risk factors, parental BMI and sociodemographic characteristics. Eur J Clin Nutr 67, 115–121. 23232586 10.1038/ejcn.2012.176

[20] Kunesova M, Vignerova J, Parizkova J, (2011) Long-term changes in prevalence of overweight and obesity in Czech 7-year-old children: evaluation of different cut-off criteria of childhood obesity. Obes Rev 12, 483–491. 21457181 10.1111/j.1467-789X.2011.00870.x

[21] Valdes Pizarro J & Royo-Bordonada MA (2012) Prevalence of childhood obesity in Spain: National Health Survey 2006–2007. Nutr Hosp 27, 154–160. 22566315 10.1590/S0212-16112012000100018

[22] Gyamfi D, Obirikorang C, Acheampong E, (2019) Weight management among school-aged children and adolescents: a quantitative assessment in a Ghanaian municipality. BMC Pediatrics 19, 376. 31651289 10.1186/s12887-019-1772-4 PMC6813048

[23] Ivanovitch K, Keolangsy S & Homkham N (2020) Overweight and obesity coexist with thinness among Lao's urban area adolescents. J Obes 2020, 5610834. 32864170 10.1155/2020/5610834 PMC7444367

[24] Esmaili H, Bahreynian M, Qorbani M, (2015) Prevalence of general and abdominal obesity in a nationally representative sample of Iranian children and adolescents: the CASPIAN-IV study. Iran J Pediatr 25, e401. 26199707 10.5812/ijp.25(3)2015.401 PMC4505989

[25] Silva APD, Feilbelmann TCM, Silva DC, (2018) Prevalence of overweight and obesity and associated factors in school children and adolescents in a medium-sized Brazilian city. Clinics (Sao Paulo) 73, e438. 30517282 10.6061/clinics/2018/e438 PMC6238815

[26] Dumith SC & Farias Júnior JC (2010) Overweight and obesity in children and adolescents: comparison of three classification criteria based on body mass index. Rev Panam Salud Publica 28, 30–35. 20857018 10.1590/s1020-49892010000700005

[27] Hedley AA, Ogden CL, Johnson CL, (2004) Prevalence of overweight and obesity among US children, adolescents, and adults, 1999-2002. JAMA 291, 2847–2850. 15199035 10.1001/jama.291.23.2847

[28] Lobstein T & Frelut ML (2003) Prevalence of overweight among children in Europe. Obes Rev 4, 195–200. doi:10.1046/j.1467789X.2003.00116.x. 14649370

[29] Taleb S & Agli A (2009) Obesity of the child: role of the socio-economic factors, parental obesity, food behavior and physical activity in schoolchildren in a city of east Algeria. Cah Nutr Diet 44, 198–206.

[30] Olumakaiye MF (2008) Prevalence of underweight: a matter of concern among adolescents in Osun State. Pakistan J Nutr 7, 503–508.

[31] Karki A, Shrestha A & Subedi N (2019) Prevalence and associated factors of childhood overweight/obesity among primary school children in urban Nepal. BMC Public Health 19, 1055. 31387571 10.1186/s12889-019-7406-9 PMC6685156

[32] Górnicka M, Hamulka J, Wadolowska L, (2020) Activity-inactivity patterns, screen time, and physical activity: the association with overweight, central obesity and muscle strength in Polish teenagers. Report from the ABC of healthy eating study. Int J Environ Res Public Health 17, 7842. 10.3390/ijerph17217842 PMC7662883 33114707

[33] Adebimpe WO (2019) Prevalence and knowledge of risk factors of childhood obesity among school-going children in Osogbo, south-western Nigeria. Malawi Med J 31, 19–24. 31143392 10.4314/mmj.v31i1.4 PMC6526345

[34] Garrido-Miguel M, Cavero-Redondo I, Álvarez-Bueno C, (2019) Prevalence and trends of overweight and obesity in European children from 1999 to 2016. JAMA Pediatr 173, e192430. 31381031 10.1001/jamapediatrics.2019.2430 PMC6686782

[35] Hoang NTD, Orellana L, Le TD, (2018) Anthropometric Status among 6–9-year-old school children in rural areas in Hai Phong City, Vietnam. Nutrients 10, 1431. 10.3390/nu10101431 PMC6212902 30287764

[36] Camelo Ldo V, Rodrigues JF, Giatti L, (2012) Sedentary leisure time and food consumption among Brazilian adolescents: the Brazilian national school-based adolescent health survey (PeNSE), 2009. Cad Saude Publica 28, 2155–2162. 23147957 10.1590/s0102-311x2012001100015

[37] De Souza Dantas M, Dos Santos MC, Lopes LAF, (2018) Clustering of excess body weight-related behaviors in a sample of Brazilian adolescents. Nutrients 10, 1505. 10.3390/nu10101505 PMC6213791 30326590

[38] Lopez-Gonzalez D, Partida-Gaytán A, Wells JC, (2020) Obesogenic lifestyle and its influence on adiposity in children and adolescents, evidence from Mexico. Nutrients 12, 819. 10.3390/nu12030819 PMC7146202 32204522

[39] Mihrshahi S, Drayton BA, Bauman AE, (2017) Associations between childhood overweight, obesity, abdominal obesity and obesogenic behaviors and practices in Australian homes [published correction appears in *BMC Public Health*. 2017 Sep 22;17 (1), 736]. BMC Public Health 18, 44. 28732475 10.1186/s12889-017-4595-y PMC5521098

[40] El-Gilany AH & El-Masry R (2011) Overweight and obesity among adolescent school students in Mansoura, Egypt. Child Obes 7, 215–222.

[41] Ghobadi, S, Totosy de Zepetnek, JO, Hemmatdar, Z, (2018) Association between overweight/obesity and eating habits while watching television among primary-school children in the city of Shiraz, Iran. Public Health Nutr 21(3):571–579. 29173231 10.1017/S1368980017003251 PMC10260791

[42] El-Kassas G & Ziade F (2017) Exploration of the risk factors of generalized and central obesity among adolescents in north Lebanon. J Environ Public Health 2017, 2879075. 29056975 10.1155/2017/2879075 PMC5615952

[43] Leal VS, Lira PI, Oliveira JS, (2012) Overweight in children and adolescents in Pernambuco State, Brazil: prevalence and determinants. Cad Saude Publica 28, 1175–1182. 22666821 10.1590/s0102-311x2012000600016

[44] Marques GFS, Pinto SMO, Reis ACRDS, (2021) Adherence to the Mediterranean diet in elementary school children (1st cycle). Rev Paul Pediatr 39, e2019259. 32785430 10.1590/1984-0462/2021/39/2019259 PMC7409091

[45] Sirico F, Fernando F, Bianco A, (2020) Parental perception of children's weight status: love overpasses scientific evidence! A cross-sectional observational study. High Blood Press Cardiovasc Prev 27, 29–34. 31802420 10.1007/s40292-019-00352-2

[46] Tapia Serrano MA, Vaquero-Solís M, López-Gajardo MA, (2021) Adherence to the Mediterranean diet, and importance of physical activity and screen time in Extremaduran High School adolescents. Nutr Hosp 38, 236–244. 33319582 10.20960/nh.03372

[47] Novak D, Štefan L, Prosoli R, (2017) Mediterranean diet and its correlates among adolescents in non-Mediterranean European countries: a population-based study. Nutrients 9, 177. 10.3390/nu9020177 PMC5331608 28241432

[48] Conde WL, Mazzeti CMDS, Silva JC, (2018) Nutritional status of Brazilian schoolchildren: National Adolescent School-based Health Survey 2015. Rev Bras Epidemiol 21, e180008. 10.1590/1980-549720180008.supl.1 30517459

[49] Bahreynian M, Qorbani M, Khaniabadi BM, (2017) Association between obesity and parental weight status in children and adolescents. J Clin Res Pediatr Endocrinol 9, 111–117. 28008863 10.4274/jcrpe.3790 PMC5463282

